# Time Perception in Adult ADHD: Findings from a Decade—A Review

**DOI:** 10.3390/ijerph20043098

**Published:** 2023-02-10

**Authors:** Christian Mette

**Affiliations:** Department of Psychology, Immanuel-Kant-Str. 18–20, Protestant University of Applied Sciences, 44809 Bochum, Germany; mette@evh-bochum.de

**Keywords:** adult ADHD, time perception, time estimation, time reproduction, time management, duration discrimination, narrative review

## Abstract

Time perception is impaired in adult ADHD. Since the term time perception subsumes different constructs, including time estimation, time reproduction, time production, and duration discrimination, it remains open whether certain domains are more affected than other domains in adult ADHD. The aim of this explorative review is to present the current state of research on time perception in adult ADHD by analysing studies from the past 10 years. A review of the literature addressing adult ADHD time perception, time estimation, and time reproduction was performed. The search strategy was conducted by using the databases “PubMed”, “Medline”, and “PSYNDEX”. The results of the present review indicate that the number of studies on time perception in adult ADHD is very scarce. Moreover, the main investigated domains of time perception in the past decade were time estimation, time reproduction and time management. Whereas some of the found studies were able to demonstrate a distinct deficit in time estimation, time reproduction and time management other studies were unable to demonstrate a clear association between ADHD and time estimation and time reproduction deficits. However, the diagnostic protocols, study design, and methodology varied between studies. Further studies on time estimation and time reproduction need to be carried out.

## 1. Introduction

Attention deficit/hyperactivity disorder (ADHD) is a mental disorder in children and adolescents and can persist into adulthood [1,2]. It is characterised by developmentally inappropriate inattention, motor hyperactivity, and impulsivity, with difficulties in all areas of daily life [3,4,5,6]. Longitudinal studies were able to show that these figures increase up to 78% if the patients with partially remitted symptoms were also included [7,8]. In an international examination of the prevalence of adult ADHD, an analysis of the WHO World Mental Health Survey in 20 countries reports a prevalence of 2.8% [9,10]. In an analysis of meta-analyses of the last 15 years that was conducted by the World Federation of ADHD, the worldwide prevalence of ADHD ranged from 2.2% to 5.9%. The prevalence of adult ADHD in Germany is approximately 3.1%, with a sex ratio reported between 2:1 (male/female) and 1.6:1 [1,10,11,12]. About 50%–60% of children also suffer from the symptoms of ADHD in adulthood [9]

However, studies have identified various aetiology factors [3,13,14]. These include genetic and environmental factors like smoking during pregnancy, stress, infection, poverty, trauma, and exposure to toxicants [15,16,17,18,19,20] that can lead to changes in brain structure, catecholaminergic neurotransmission, and to specific symptoms like inhibitory and attentional deficits [21,22,23,24,25,26,27]. Recent studies have demonstrated that ADHD is associated with various cognitive dysfunctions and structural changes [6,28,29,30,31]. A study on the neuropsychological domains showed that patients with ADHD had deficits in sustained attention, working memory, variability of reaction times, and delay aversion [32]. Furthermore, there is a higher inter-individual variability in cognitive domains, and up to 70% report more than one affected cognitive domain [33]. Similarly, from a clinical perspective, a problem in the neuropsychology of ADHD is that there are no standard neuropsychological models for ADHD in adulthood. An early assumption on the aetiology of the cognitive deficits postulates that the symptomatology arises due to a lack of behavioural inhibition, which in turn leads to executive problems in patients [34,35,36]. Considering a single domain is not sufficient and additional studies could show heterogeneity in terms of cognitive dysfunction in ADHD. In this context, multi-deficit models now dominate [37]. In contrast, the dual-pathway model refers to deficits in cognitive inhibition and motivational factors as the basis of neuropsychological heterogeneity in ADHD [37,38]. However, this model was upgraded to a Triple-Pathway Model and identifies the changes in time perception as an additional neuropsychological dimension of ADHD [39]. Moreover, studies investigated the biological pathways and the related symptoms of children and adolescents. The studies demonstrated separable cognitive components, including deficits in cognitive control, reward processing, and timing deficits [40]. Those results converge the findings of Sonuga-Barke regarding a timing deficit in ADHD.

In the consideration of time or the perception of time, early neuropsychological studies demonstrated that time is often perceived as a “flow” and “time” is of an essential role in functions such as walking, music, sports, or the day–night rhythm in everyday life [41,42]. Since time or the “flow of time” is never perceived “directly” but by changes within an individual reference system, the human organism needs an internal subjective time system to measure “objective time” without the assistance of external cues [43,44]. In the literature, three different timing concepts are postulated—“circadian timing”, “interval timing”, and “milli-second timing”. Circadian timing, also known as the internal clock, has the function of regulating periodic bodily functions such as temperature, blood pressure, neurophysiological and physiological activity levels in a 24-h cycle. The nucleus suprachiasmatic, which is located in the ventral hypothalamus, has been identified as the neuroanatomical correlate of the internal clock which synchronises with the input of daylight and the neurotransmitter melatonin 24-h rhythm in mammals [45].

Interval timing is characterised as the organism’s ability to track the passage of time in the seconds to minutes range. Interval timing is involved in decision-making and conscious time estimation, whereas the perception of the milliseconds is important for motor processes, speech, and music perception [43,44]. The perception and the decision-making of time intervals, as well as cognitive processes, are closely interconnected. This was also shown in early studies concerning animal and human time perception and brain lesions studies. It was demonstrated that the cerebellum, the basal ganglia, the hippocampus, and the prefrontal cortex are crucial for the perception of time [46,47,48,49]. Furthermore, research indicates that thalamo-corticalstriatal circuits are activated during timing tasks. These circuits include the structures of the basal ganglia, the prefrontal cortex, and the posterior parietal cortex [50,51]. Functional MRI studies in adult ADHD showed decreased activity in the temporo-occipital and prefrontal cortices, which are responsible for time and memory processing [52,53,54]. It is supposed that a central timer is located in the cerebellum, whereas the prefrontal cortex mediates the planning abilities, operating the perception of intervals [55,56].

Therefore, time perception is a multidimensional structure that encompasses different cognitive and neuropsychological functions. These functions can be affected in adult ADHD. Hence, research on time perception in adult ADHD needs to be analysed in more detail. Previous research assumes that different domains are affected in the context of timing in ADHD. These are the temporal organization of motor behaviour (motor timing), the ability to estimate time intervals (perceptual timing), and the ability to anticipate the consequences of a decision (temporal foresight) [57]. Thus, different types of methods are used to investigate the perception of time, which are described in the basic literature. These are time estimation tasks, time production tasks, time reproduction tasks, and duration discrimination tasks. The ability to estimate time is of clinical relevance because it is one of the most important neuropsychological functions on which many daily activities depend. Time estimation (TE) involves estimating the duration of a completed (retrospective) or an upcoming interval or period (prospective) in order to make decisions. In ADHD, this ability is also of importance in everyday tasks such as scheduling, planning activities, and in social interactions. The patients often show deficits in this area. Thus, time deficits become critical when patients return home from a clinical setting or when they start/restart work. As time estimation affects other cognitive domains that are impaired in ADHD, it is even more important to identify the exact mechanisms to derive and develop therapeutic tools that can support the affected patients. Time reproduction (TR) refers to the reproduction of a time interval. The constructs of TE and TR are artificially separated, but they should always be considered together when evaluating results. TR tasks require participants to reproduce longer time intervals (2–40 s) [57], and a differentiation between retrospective and prospective time perception tasks has to be made Nevertheless, it can be assumed that TE refers to the estimation of the duration between two events and TR refers to the reproduction of the duration between two events. From a clinical perspective, it is apparent that this ability could also play a role in the planning ability of patients with ADHD. From a clinical perspective, the question remains as to why patients are noticeably late for appointments and whether a deficit in time reproduction, memory processes, or another cognitive domain might be responsible for this. Moreover, motor timing tasks often examine the performance of participants in the short-second range by using tasks like a tapping task [58,59]. TR tasks require participants to reproduce longer time intervals (2–40 s) [57] and a differentiation between retrospective and prospective time perception tasks has to be made [59]. In time production experiments, the participants produce short or long time intervals. [41,42]. These tasks demand different cognitive and neuropsychological abilities than in TR and TE. Furthermore, studies also explore time management. Time management is probably one of the most discussed topics in psychology. Patients with ADHD often report problems with time management. This problem can interfere with the treatment of ADHD (e.g., tardiness, neglect, or failure to do given tasks). According to Claessens et al. (2007), time management is defined as behaviour that uses time effectively and economically in specific activities. Therefore, time management is closely related to work, academic success, job satisfaction, and to stress in ADHD [60]. However, time management is also a multidimensional construct which not only includes abilities like concentration and memory but also the whole spectrum of executive functions. Therefore, it can be seen as a complex structure in the context of time perception, and since this involves abilities that are deficient in ADHD, it is not surprising that patients with ADHD report problems in this area.

When considering studies on time perception in ADHD, it has to be stated that the results are heterogeneous due to a lack of the studies in this field of research. Studies concerning time perception in ADHD often have mixed results because differences in children, adolescents, and adults must be taken into account. In addition, various factors (e.g., symptomatology, comorbidities) play an important role in the assessment and interpretation of the studies. This makes a general interpretation and evaluation of time perception in ADHD complex. Nevertheless, recent meta-analyses on children with ADHD demonstrate that deficits in time perception are detectable [61,62]. A longitudinal study observed 158 children with ADHD over a period of more than 20 years. Different neuropsychological domains, including time perception, were examined over a course of 20 years. The results showed that children with ADHD made more errors in time reproduction. This factor remained stable into adulthood [63]. At this point, it remains unclear as to whether these results can also be applied to adult ADHD. However, an impaired time perception ability is of relevance for adult ADHD. To mention a few early studies in the area of research on time perception in adult ADHD, those studies have shown that ADHD patients have significant difficulties with reproduction and estimation of time intervals [64,65,66]. A seminal, early study also found that adult patients with ADHD have problems in the reproduction of time interval [67]. In a further, early translational study, time discrimination ability and time reproduction ability in children with ADHD, in children with dyslexia, and in adolescents with ADHD was investigated. Children and adolescents showed a deficit in time discrimination, which was reflected in the precision of the responses and in greater inter-individual variability [68]. A meta-analysis of studies with children with ADHD examined data from 11 fMRI studies with a total of 150 adult ADHD patients and 145 healthy controls. The results show consistent and replicable deficits in the brain areas closely associated with timing. These include the cerebellum, left inferior prefrontal cortex, and left inferior parietal lobes. Furthermore, areas are activated that are related to problems with the deactivation of the default mode network (precuneus, posterior cingulate). The findings also show that a long-term medication in adult ADHD could have an essential influence on the functionality of the right dorsolateral prefrontal cortex (DLPFC). The meta-analysis also shows that the inferior prefrontal cortex, together with the insula, plays a role in subsecond and suprasecond motor and perceptual timing. In addition to that, the results highlight the role of the cerebellum in temporal predictions, with the medial cerebellum being involved in visual timing. Interestingly, the meta-analysis did not show a deficit in the basal ganglia [69].

However, it remains unclear whether there is a specific domain of time perception that might be more affected in adult ADHD (e.g., time estimation). In addition, the subdomains of time perception also require different neuropsychological capacities. Thus, it remains unclear whether the neuropsychological deficits and/or the symptomatology also contribute to time perception abilities in adult ADHD. In addition, a possible methodological impact should be considered in all studies of time perception in ADHD. Finally, potential changes in time perception in the transition from childhood to adulthood need to be considered. Therefore, the focus of this review was on adults, as there is less research on time perception compared to children with ADHD. In addition, there is no review that focused on the domains of time perception in adult ADHD. This implies a structured presentation of the research on time perception in adult ADHD. Moreover, there is also a diversity of methods in the field of time perception assessments in ADHD, because there are no validated and standardized time perception assessments available. Therefore, the scope of this explorative review was to describe the methodology (target (e.g., time estimation) aim, design, outcome) and issues in the context of time perception in adult patients with ADHD. Nevertheless, it remains unclear whether adult patients with ADHD have a general deficit in the described domains of time perception or whether this effect is influenced by cognitive deficits in adult ADHD. Thus, the aim of this explorative review is purposely limited to present the current state of research on time perception in adult ADHD by analysing studies from the past 10 years.

## 2. Methods: Literature Search

The rationale of the study was to present the current state of research on time perception in ADHD with a focus on the approach (e.g., time estimation, time reproduction), the methodology (e.g., timing task, interval timing), and the outcome. An explorative review was conducted. Here, a structured review of the literature addressing adult ADHD and time perception was performed. The review examines the domains of time perception in adult ADHD. It employs a narrative explorative structured method with the following search criteria, which were derived from literature: The search strategy was conducted by using the databases “Pubmed”, “medline”, and “PSYNDEX”. The mentioned databases were searched from January 2022 to October 2022. The following search terms were defined: “adult ADHD”, “attention deficit hyperactivity disorder”, “adults”, “time”, “timing”, “timing deficit”, “time deficit”, “temporality”, “time perception”, “time estimation”, “time reproduction”, “time production”, “duration discrimination”, “time management”, and “time processing”. Moreover, “and” was defined as boolean operators (see Supplementary Online Material.

In addition, according to the literature, the following variables were derived and defined as inclusion criteria for the present review: firstly, papers had to be written in English or German and had to be listed in the databases “PubMed”, “Medline”, and “PSYNDEX”. Secondly, original research articles, clinical trials, and meta-analyses were included in the present review. Thirdly, a period from 2012–2022 was defined as the range for the literature search. Further, adults with ADHD (>18 years) with a primary diagnosis of ADHD according to DSM-IV, DSM-5, or ICD-10 were included. Neither ADHD subtype presentation, gender/sex, comorbidities, nor IQ restricted the search strategy. The following exclusion criteria were derived from the literature and defined for the present review: studies not including ADHD and time perception as described under inclusion criteria, studies including only children and adolescents, publications not subject to peer review, non-English or German papers, case studies, reviews, studies describing only psychiatric comorbidities, and pharmacological trials and studies older than 10 years. The results are described in a descriptive table according to the applied study design. In addition, if effect sizes were not reported in the original paper, effects sizes were calculated when possible by using Cohen’s d (small effects (d ≤ 0.2), medium effects (d ≥ 0.5), and large effects (d ≥ 0.8)) [70].

## 3. Results

**Figure 1:**
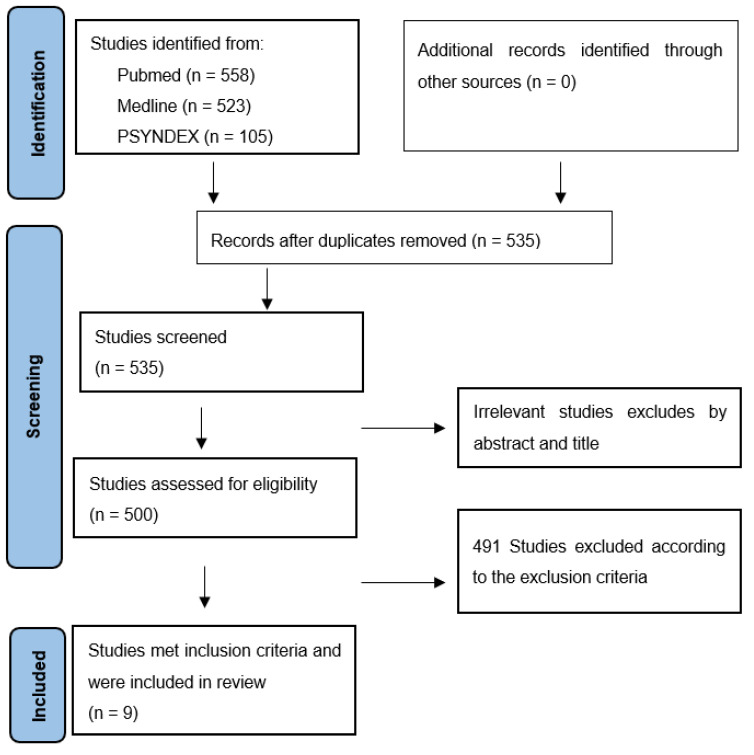
PRISMA flow chart article disposition.

After removing duplicates and studies published prior to 2012, all studies were screened by title and abstract, excluding papers that clearly did not fulfil the inclusion criteria. Furthermore, reference lists of the retrieved papers were hand-searched to identify additional relevant articles. Other papers of interest found in the manual search published January 2012 to December 2022 were also included. In total, n = 535 papers were assessed in full text. After checking for inclusion and exclusion criteria, only n = 9 articles were included (see Figure 1). However, there was a profound variation in the characteristics of the study samples of the reviewed articles (e.g., medication use, comorbid disorders, control conditions, sample size), the type of functions assessed, the availability of outcome statistics, the type of neuropsychological tests applied, and the statistical comparisons performed. Therefore, it was deemed unjustified to compare effect sizes across the found studies.

A first finding reveals that in all studies surveyed, the term “time perception” is equated on the one hand with time estimation and duration discrimination and on the other hand with time reproduction, time processing, and time production. The results show that the number of studies in the field of time perception in ADHD in the past 10 years is very scarce. A range of different time perception concepts were employed in connection with ADHD in adults. Moreover, the diagnostic protocols and quality of the clinical assessments of ADHD varied between studies. Nevertheless, these terms imply different constructs in the research of time perception in ADHD and hence require to be described, interpreted, and evaluated separately from one another. Due to the limited number of studies on the topic of time perception, studies are presented according to the hypotheses, the sample, the method, and the results (see Table 1). In addition, due to the limited number of studies found on the subject, these studies will be analysed in more detail (see above).

Taken together, three studies used behavioural design, three studies used imaging techniques, one EEG study was available, and one RCT was conducted to investigate time perception in ADHD. In the analysis of methodology and design, all studies showed a sufficient level of evidence regarding the applied methodology. Only one study did not have a control group, whereby this limitation was compensated by a crossover design. Four studies investigated time estimation, four studies investigated time estimation, and one study investigated time management.

### 3.1. Time Estimation in Adult ADHD

The experimental designs, approaches, and results of the found studies on time estimation (TE) are heterogeneous. Firstly, an fMRI study investigated the impact of distractors on brain activity and performance in a TE task in adults with ADHD [71]. The fMRI analysis showed a higher activation of the superior orbitofrontal gyrus in the distractor condition in adult ADHD. Further, the results show that the “difference between conditions” is a predictor of neuronal activity in the right medial frontal gyrus, the right thalamus, and the cerebellum [71].

A further study examined the exposure to a TE task and the effect of the exposure on the theta band activity in the dorsolateral (DLPFC) and the ventrolateral prefrontal cortex (VLPFC) [72]. Theta waves are important in aspects of cognition and behaviour. These include learning and memory [80]. The findings of the study indicate that the exposure for 30 days enhances the cognitive attentional levels in the patients with ADHD and causes a decrease in the EEG theta power. The results also show that the absolute error was higher in the experimental condition compared to controls for all time intervals. The study stated that the decrease in the EEG theta power might indicate an accumulation of temporal pulses. Thus, this could be responsible for the improvement of the cognitive abilities in adult ADHD [72]. In contrast, a further study investigated different neuropsychological domains (EF, reward, and timing). However, a time interval of only one second was used, and the study reported no significant differences between adult ADHD and healthy controls. In this context, the short duration of the time interval in the TE task might be an explanation for the results [73]. In a Magnetoencephalography (MEG) study, the cortical networks underlying temporal perception (PFC, cerebellum, basal ganglia, SMA, and ACC) were investigated. The aim of the study was to investigate neural gamma activity in a TE paradigm in adult ADHD [74]. The results show that unmediated adult patients with ADHD showed a higher error rate in the TE task compared to the healthy control group. Furthermore, the study showed that adult ADHD patients without medication have significantly reduced activity in the gamma-frequency spectrum. In addition, the study showed that these gamma deficits were found in the ROI areas of the right anterior cortices, left anterior cortices, supplementary motor area, left prefrontal cortex, and ACC. Besides, the study showed that the participants with medication had a low error rate in the TE task and an increase in gamma activity [74]. Further, the study demonstrates that gamma activity is impaired in the neuroanatomical areas of time perception. This is a further indication that the problems of time estimation in adult ADHD are also observed on a neurofunctional level. Finally, the study also showed an effect of medication. This is of crucial importance for treatment and emphasizes that ADHD is a multidimensional disorder.

### 3.2. Time Reproduction in Adult ADHD

When considering the research on TR in adult ADHD, it is worth noting that only four studies were published in the last 10 years. Moreover, the experimental designs, approaches, and results of the found studies on TR are also heterogeneous. First, Marx et al. (2013) investigated the motivational effects of financial rewards on cognitive function profiles in adult ADHD [78]. In this study, the domains of inhibition, working memory, sustained attention, delay aversion, time discrimination, and time reproduction were examined. Results indicate that reward minimized the discrepancy scores in adult ADHD and balanced the effect of group between the participants. A further study examined the effect of working memory (WM) and short-time memory (STM) on time reproduction performance [77]. The results show no group difference in the TR task with the exception for a higher variability in 1 s, 4 s, and 6 s for unmedicated patients with ADHD and at the 4 s and 6 s interval in medicated patients with ADHD. Besides, no association between WM and TR was detected [77]. Another study investigated TR and its neuroanatomical correlates in adult ADHD and unaffected first-degree relatives [76]. The study found a positive correlation between the grey-matter volume in the cerebellum and the performance in TR. Accordingly, the more grey-matter volume that was in the right lobule V, the poorer the performance was in the TR in the sample, making these findings significant for ADHD. For the other ROIs, no effect was detected. The authors suggest that the involvement of the cerebellum, rather than the basal ganglia, or prefrontal cortex is linked to the encoding phase and is not related to the deficits in maintaining and monitoring temporal information in working memory. Moreover, they argue that grey matter abnormalities are responsible for problems in the frontocerebellar networks, which are crucial for time processing [76]. A recent study evaluated the auditory and visual TR in adult ADHD [75]. Furthermore, the study sought to investigate the involvement of working memory, ADHD symptoms, and hyperfocusing on the performance in TR. The study found a reduced TR in adult ADHD with no modality effect. Moreover, the study detected a difference in adult ADHD with a higher discrepancy for longer intervals. In addition, TR was associated with impulsivity/hyperactivity, and no effect of WM was observed [75].

### 3.3. Time Management in Adult ADHD

However, the literature search could detect only one clinical study, which highlighted the obstacle of time management in adult ADHD. The study investigates the efficacy of a group cognitive training in time management in adult ADHD [79]. The study pointed out that an executive and a dysfunction in inhibition are closely connected to deficits in time management and time processing. Therefore, the study sought to investigate the therapeutic effects of a cognitive therapy program with a focus on time management on ADHD symptoms. The results showed that the cognitive training had an impact on all scales of the questionnaires and resulted in an improvement in the performance of patients of the intervention group.

## 4. Discussion

The results of the narrative review showed that adult patients with ADHD have problems in the estimation of time intervals, in the reproduction of time intervals, and in the management of time. In addition, the results show that these behavioural deficits can also be found at the neuropsychological level in adult ADHD. Thus, the cerebellum and the VLPFC and DLPFC are particularly relevant. Nevertheless, when analysing the available studies focused on time perception in adult ADHD, it becomes evident that only a few studies have been published in the last 10 years. This is surprising, since an impaired time perception is postulated in the triple pathway model [37,39]. When considering the studies in the context of time perception, the different results and limitations also need to be critically considered. The results of the present review indicate that whereas some studies were able to demonstrate a distinct deficit in time perception [71,72,74,75,76] other studies were unable to demonstrate a clear association between ADHD and time perception deficits [73,77,78]. In the context of TE studies, Pretus et al. (2020) could detect an effect on the distractor. However, the anticipated direction of the distractor effect was opposite. Both groups performed better when the distractor was presented. As expected, the findings of the study demonstrated the impairment of the specific neuroanatomical areas involved in the perception of time in adult ADHD. However, no difference was found between the groups in terms of TE at the behavioural level. Nevertheless, the results also show an interesting aspect of the feasible compensation of neuropsychological deficits in adult ADHD. This is also frequently observed in clinical settings, as patients are quite capable of maintaining attention over a longer period in the face of a high stimulus input. The salience of the distractor can, in turn, also increase the valence of that distractor and hence increase the dopaminergic level in the patients. The connection between valence, salience, and dopamine has already been demonstrated in a study [81]. This effect was already demonstrated in earlier studies [82] and might therefore offer a potential future research topic. Furthermore, it remains unclear how the activation of brain areas is related to the results of the behavioural outcomes [71]. Thus, the results also provide additional opportunities for research in this context.

The study conducted by Fontes et al. (2020) reports that the exposure reduced the level of inattention and impulsivity [72]. In summary, the results suggest that exposure to a TE experiment reduces theta band activity and thus improves cognitive functioning in ADHD. However, it remains to be clarified whether the ability to estimate time is also improved. Furthermore, the study also reports several limitations such as the lack of a sufficiently large sample size, the non-homogeneity, non-EEG uptake during the experiment condition, the non-association with sub-second level tasks, and the lack of a healthy control group [72]. In that study, it might also be noted that the study design could also be interpreted as a neurocognitive training that covers multiple impaired neuropsychological domains in ADHD. This might explain the effects as training effects and thus needs to be considered as a possible confounding variable [83]. Therefore, the question that remains is whether the effect is an ADHD-specific effect or a general training effect. Thus, the results of the study should be examined by further studies that take the limitations into account.

Mostert et al. (2015) stated that the short duration of the time interval in the TE task is an explanation for the results [73]. The TE task was part of a large neuropsychological test battery, yet it included only one time interval. The test battery was able to show the neuropsychological deficits in ADHD, although it was not able to show a deficit in time perception. However, this was due to the application of a single time interval. A study showed that one time interval is not sufficient for investigating a possible deficit in TE [84]. In terms of limitations, Wilson et al. (2013) pointed to the small sample size, the long duration of the intervals, the training aspects, and the lack of a placebo control group in their study [74].

In the context of studies on TR, the study by Marx et al. (2013) points to the high functional level of ADHD patients as a limitation, which might have influenced the results. Moreover, the rate of comorbid disorders in that sample was small. The authors elicited that this might be an indication as to why a large proportion of the cognitive problems in adult ADHD are mediated by the comorbid disorders [78]. Additionally, due to the lack of randomisation, order effects cannot be entirely ruled out in the study. In contrast, Mette et al. (2015) were unable to detect a clear difference in TR in adult ADHD. However, this study examined raw scores in comparison to others, whereas other studies examined absolute discrepancy scores. This could lead to different results of the found studies. Furthermore, it should be mentioned that the effects of counting and the use of external factors was highlighted as a distinct limitation [77]. Studies investigating this effect in adult ADHD have not been published. As of yet, only one previous study investigated the effect of counting on time perception in children with ADHD. The study was able to demonstrate that counting improves the children’s perception of time [85]. Hence, it can be assumed that counting or the use of external cues might be a confounding variable in time experiments. The exact mechanisms of counting in time experiments in adult ADHD have not been investigated yet and should be the subject of future studies. Pironti et al. (2016) stated that an interpretation regarding the sole contribution of the cerebellum to the TR problems needs to be discussed carefully, as the study refers to VBM data and not to fMRI data [76]. The study by Dölek et al. (2021) stated the low sample size and low number of trials in the TR task as limitations. Another limitation is the high number of male participants in the ADHD group compared to the control group [75]. Additionally, the effect of sex should be given greater consideration in future studies, since a study found that women with adult ADHD more often fall off the diagnostic grid or receive less treatment despite suffering from severe symptoms [86]. Women were also underrepresented in the studies of this review on time perception. Therefore, a sex-specific effect cannot be excluded and should be investigated in future studies.

The different outcomes of the studies show that there is still a need for research in this area. Nevertheless, it can be assumed that WM and the ability to reproduce time intervals are closely connected. Therefore, it can be derived that an improvement in working memory performance could also lead to an improvement in TR. This, in turn, would strengthen the importance of neuropsychological trainings in the treatment of ADHD. This is also an important finding for clinical practice, as enhancing a specific deficit through training might also have an impact on other neurocognitive deficits in adult ADHD.

One study examined the effect of training on time management in adult ADHD. The study found a positive effect of training on time management [79]. Furthermore, clinicians report that patients with ADHD often report problems in maintaining appointments and/or agreements. These problems lead to a high level of psychological stress. This leads to a reduced coping ability to deal with everyday life obstacles, as well as problems in social relationships in adult ADHD [87]. When analysing the results of the Japanese study [79], it can be noted that the study did not investigate time management or time perception skills. The conducted training might lead to an improvement in ADHD symptoms, which in turn leads to an improvement of time management skills. Moreover, the study stated that the effect of medication was not controlled, and participants had a high intellectual level compared to previous studies. A further limitation of the study is that there was no baseline survey of performance in time management [79]. Therefore, the study lacks an analysis of possible correlations of the individual cognitive performances (e.g., impulsivity) on time management. A follow-up study using a structural equation model to show the possible interactions would be worth investigating.

Due to the limited number of studies, general problems or open questions can only be addressed to a limited extent and the result that there are only a few studies about time perception must be regarded as a result and therefore has to be discussed: each study had strengths and limitations. Studies that have demonstrated a deficit in TE or TR have examined absolute/relative differences, discrepancies, or variability in participants’ performance [71,72,75,76], whereas other studies reported no effects. These differences in findings might be due to differences in the analysis of participants’ performance in time perception tasks. When analysing the effect sizes, it also becomes apparent that there is heterogeneity in this regard, as effect sizes ranging from small to large are calculated and observed. However, not all studies report the effect sizes, and the different methodological instruments make it difficult to interpret the studies in terms of effect sizes.

Operationalizing time perception in adult ADHD is certainly one of the major challenges. As a result, the research revealed estimation, reproduction, production, and duration discrimination in the context of time perception. However, there is a lack of validated instruments to measure time perception in ADHD. Future research should therefore develop validated measurement instruments for time perception, similar to the neuropsychological test batteries for ADHD in order to verify the previous results. A further general problem in adult ADHD is the presence of comorbidities. Only one of the found studies reported no comorbid disorders in their sample [71]. This applies not only to time perception studies but to all clinical and neuropsychological research in adult ADHD. A recent study has already shown that there is a high level of comorbid disorders in children with ADHD [88]. Studies have also shown that there is a connection between time perception and various mental disorders [89]. Thus, it cannot be excluded that the presence of comorbid disorders in ADHD might also explain the heterogeneity of the study results on time perception. Therefore, the impact of comorbidities should be taken into account in future studies, e.g., by considering clinical control groups.

Moreover, an explanation for the limited number of studies in this topic is probably due to the complexity of the concept time perception. A large number of research concerning time shows that the ability to perceive time is also dependent on higher cognitive performance [41,42,43,44,45,46,47,48,49,52,53]. Hence, it seems logical that a disorder that is associated with problems in higher cognitive performance also shows problems in time perception. However, the research in the context of time perception lacks a specific aspect. It is not sufficiently clear how the symptoms of ADHD such as EF, delay aversion, memory, impulsivity, and the ability to reproduce time, to estimate time, and to discriminate duration are related. Therefore, future research should highlight and analyse the interaction between time perception and higher cognitive abilities by employing a structural equation model. Future research must clarify whether the variability has an impact on the results of the studies or whether other variables might be involved.

Finally, it is also of interest that there is no study on time production. An explanation for this can, at most, be speculated and not adequately validated. Hypothetically, there might be an overlap with motivation research. Nevertheless, future studies should also investigate the role of time production in ADHD.

## 5. Conclusions 

The experimental results of the studies found are very valuable in understanding ADHD and time perception. A distinct problem in TE, TR, and time management could be revealed in most of the studies, leading to the assumption of a general time perception deficit in adult ADHD. Therefore, these results support the postulated findings of the triple pathway model. However, it remains unclear whether the “real-life” experience of the patients can be represented in its entirety. The results clearly demonstrate that time perception in adult ADHD has a very high complexity and variability.

Hence, to provide a transfer to clinical treatment, the following issue might be relevant for clinicians. The findings of the studies not only indicate a deficit in time perception, but also in terms of the contribution of different neuropsychological domains and structures (e.g., memory). Therefore, an enhancement of the functionality of these domains could also have an impact on time perception in adult ADHD. However, the treatment manuals designed for adults with ADHD do not consider a deficit in time perception. Therefore, the treatment of adult ADHD should also focus more on the problems of time perception. This, in turn, could sustainably improve various psychosocial problems (e.g., conflicts due to tardiness, planning ability) and the quality of life of people with ADHD. There are other topics that were not addressed in the current review and need to be investigated in future studies, like OTMP, and the neurobiology of time deficits in adult ADHD. Operationalizing TE and TR is highly challenging, and many issues are unsolved. Finally, adult patients with ADHD have problems in the estimation of time intervals, in the reproduction of time intervals, and in the management of time. Due to a lack of publications on this topic, the underlying behavioural findings that contribute to time perception in adult ADHD (perception, attention, working memory, action planning) should undergo further critical examination. Nevertheless, research on time perception remains a fascinating topic for future studies and it might contribute to significant developments in terms of understanding and treating the disorder.

## Figures and Tables

**Table 1:** Sample characteristics and results of reviewed studies (n = 9).

Study	Target	Aim	Sample	Age (Mean, SD)	Design	Outcome	Effect Sizes
Pretus et al. 2020 [71]	Time estimation, fMRI	impact of distractors on brain activity and performance in a time-estimation task	N = 21 (ADHD, combined subtype; 10 female) N = 24 (healthy controls, 12 female) ADHD Patients met DSM-IV criteria No comorbidity	ADHD: m = 36.48 (6.90) Healthy controls: m = 34.33 (7.73) Range = 19–50 (whole sample)	Behavioural design: estimate intervals 1–6 s; In 50% of the trial’s presentation of a distractor (Moving element) Measures fMRI (ROI): ACC, DLPFC ANOVA, T-test	Behavioural data: time estimation error: n.s. absolute difference distractor vs. non-distractor: (*p* = 0.032) within-group effect distractor ADHD < Controls (*p* = 0.003) interaction distractor vs. group: n.s. Whole brain analysis: Distractor vs baseline ADHD > Controls: bilateral superior orbitofrontal activation (*p* = 0.019) Non-distractor vs baseline ADHD < Controls: ADHD decreased activation right pre-central, post-central (*p* = 0.027); bilateral supplementary motor area (p = 0.005). ROI analyses ACC and DLPFC: n.s.	d = 0.42 d = 0.66 d = 0.97 t = 4.35
Fontes et al. 2020 [72]	Time estimation, EEG	Exposure to a time estimation task modulates theta band activity in the DLPFC and the VLPFC. Exposure leads to a decline in symptoms in adult ADHD	N = 24 (ADHD; 16 male, 6 female) ADHD patients met ICD-10 criteria	ADHD: m = 23 (1.4) Range = 20–30	crossover experiment visual time-estimation task: 1 s, 4 s, 7 s, 9 s randomly presented experimental condition: 30 days exposure to time-estimation tasks plus EEG each week control conditions: no exposure to time estimation tasks measure: RMANOVA	Behavioural data: Experimental vs. control; ADHD > Controls Absolute error (AE): 1 s (*p* < 0.002) 4 s (*p* = 0.001) 7 s (*p* < 0.002) 9 s (*p* = 0.02) Experimental vs. control, ADHD > Controls Relative error (RE): 1 s (*p* < 0.001) 4 s (*p* < 0.0001) 7 s (*p* < 0.001) 9 s (*p* < 0.001) ADHD < Controls Regression analysis Absolute/Relative Error vs. Experimental/control *p* < 0.0001 for 7 s, 9 s (AE) and 7 s, 9 s (RE) ADHD < Controls EEG Data: RMANOVA shows interaction for conditions, cortical areas, and visits (*p* < 0.001) Interaction analyses: conditions and visits (*p* < 0.001) conditions and cortical areas (*p* < 0.001) visits, and cortical areas (*p* < 0.0001). theta band right DLPFC and VLPFC increased with thirty days of time-estimation task exposure, ADHD>HC (*p* < 0.05).	d = 0.51 d = 0.62 d = 0.94 d = 0.86 d = 0.86 d = 0.61 d = 0.94 d = 0.82 OR = 3.8 (CI = 3.4–4.3) OR = 3.3 (CI = 2.9–3.7) OR = 1.2 (CI = 1.6–2.2) OR = 4.5 (CI = 1.4–1.5) η2 = 0.13 η2 = 0.22 η2 = 0.10 η2 = 0.30 d = 0.20
Mostert et al. 2015 [73]	Time estimation EF, reward	Neuropsychological performance in adult ADHD	N = 116 (adult ADHD 42% male) N = 126 healthy controls 40% male)	ADHD: m = 35.6 (10.40) range 19–63 HC: m = 36.30 (11.75) range 19–63	Neuropsychological test battery (EF, delay discounting, time estimation) Time estimation interval 1s (Median response time, absolute median deviation) Measures: ANOVA	Behavioural data: Median response time: n.s. absolute median deviation: n.s.	D = 0.14 D = −0.30
Wilson et al. 2013 [74]	Time estimation MEG	gamma activity in cortical networks (PFC, cerebellum, basal ganglia, SMA, and ACC)	N = 12 (adult ADHD, 4 females, medicated/unmedicated) N = 12 (healthy controls, 4 female) ADHD patients met DSM-IV Criteria	ADHD: m = 41.83 range 30–58 HC: m = 40.08 range 28–62	continuous long duration time estimation task Estimation passage of time (1 min increments) 4 min recording period Measure: ANOVA, T-Test ROI: MEG gamma activity ACC, SMA, right/left anterior frontal cortices, right and left PFC	Behavioural data: ANOVA session-by-group interaction effect (*p* = 0.05) main effect of session (*p* < 0.01) group main effect (n.s.) T-Test session-by-group interaction (unmedicated vs. controls; *p* = 0.04; medicated vs controls n.s.) medication time estimation accuracy (*p* < 0.001) MEG Data: ANOVA location-by-frequency-by-group three-way interaction effect (*p* < 0.01) frequency-by-group interaction (*p* < 0.01) location-by-group interaction (*p* < 0.01), main effect of group (*p* < 0.01) post hoc t-test unmedicated ADHD vs. controls weaker low, mid, and high gamma activity (*p* = 0.01)	d = 0.83 D = 1.46 D = 0.75 D = 0.26 d = 1.5 d = 0.71 d = 1.1 d = 0.73 d = 1.2
Dölek et al. 2021 [75]	time reproduction (auditory and visual)	involvement of working memory, ADHD symptoms, hyperfocusing on time reproduction ability	N = 32 (adult ADHD, 21 males, 11 females) N= 32 (healthy controls, 9 males/23 females) ADHD Patients met DSM-5 Criteria	ADHD: m = 25.34 (6.01) HC: m = 24.90 (3.89)	Time reproduction task: eight time intervals (500 ms, 1000 ms, 2000 ms, 4000 ms, 6000 ms, 8000 ms 12,000 ms, 16,000 ms) The intervals were displayed either auditory with a white noise or visual via a displayed red circle. Measures: ANOVA, Mean Absolute Discrepancy Score (ADS), Accuracy Coefficient Score (ACS) Memory assessment: 2-back task	Behavioural data: Main effect group (*p* < 0.001) time Interval (*p* < 0.001) group x time Interval interaction effect (*p* < 0.001) ACS auditory TRT: both groups reproduced shorter durations for all time intervals ADS visual time reproduction larger ADS for all time intervals (except for 500 and 8000 ms) (*p* = 0.001–0.42) ADS auditory time reproduction task larger ADS for all time intervals (except for 500, 1000, and 8000 ms) (*p* = 0.001–0.003).	η2 = 0.23 η2 = 0.58 η2 = 0.15 r = 0.40–0.26 r = 0.65–0.37
Pironti et al. 2016 [76]	Time reproduction, VBM	time reproduction ability in adult ADHD; examine a potential neuroanatomical correlate for time reproduction in adult ADHD.	N = 20 (adult ADHD) N = 20 (non-affected first-degree relatives) N = 20 (healthy controls) ADHD Patients met DSM-IV Criteria	ADHD: m = 32.2 (10.31) Relatives: m = 38.85 (15.31) HC: m = 32.55 (5.8)	Time reproduction task seven time interval: 500 ms, 1000 ms, 3000 ms, 6000ms, 12,000 ms, 18,000 ms, 24,000 ms Measures: ANCOVA absolute discrepancy score VBM ROI: PFC, basal ganglia, cerebellum	Behavioural data: Absolute discrepancy: main effect of group (*p* = 0.009) ADHD < Controls; ADHD = relatives effect of duration (*p* < 0.001) interaction duration vs. group (*p* = 0.003) VBM data: total brain volume and total intracranial volume n.s. correlation absolute discrepancy and ROI cerebellum (r = 0.297, *p* = 0.021) other ROIs: n.s.	d = 0.56 d = 0.88 d = 0.59
Mette et al. 2015 [77]	Time reproduction	Examine the association between time reproduction and WM and STM.	N = 30 (adult ADHD, medicated) N = 29 (adult ADHD, unmedicated) N = 32 (healthy controls) ADHD Patients met DSM-IV Criteria	ADHD (medication): m = 34.73 (9.08) ADHD (unmedicated): m = 34.72 (10.40) HC: m = 31.28; (7.14)	Time reproduction task six time intervals: 1 s, 4 s, 6 s, 10 s, 24 s, 60 s Memory assessment: Wechsler memory scale Measures: Kruskal–Wallis H-test, ANOVA, time reproduction raw scores, variability, coefficient of variation	Behavioural data: Time reproduction raw scores: n.s. Variability: ADHD>HC 1 s (*p* < 0.04) 4 s (*p* < 0.01) 6 s (*p* < 0.01) Other n.s. coefficient of variation: ADHD>HC 1 s (*p* < 0.04) 4 s (*p* < 0.01) 6 s (*p* < 0.03) Other n.s. Memory: ADHD < HC (*p* = 0.03)	d = 0.44 d = 0.57 d = 0.56 d = 0.46 d = 0.54 d = 0.49 d = 0.48
Marx et al. 2013 [78]	Time reproduction, time discrimination, inhibition, sustained attention, memory, EF, delay aversion	Examine motivational effects of financial reward on cognitive functions in adult ADHD	N = 38 (adult ADHD, with/without reward) N = 40 (healthy controls, with/without reward) ADHD Patients met DSM-IV Criteria	ADHD (reward) m = 29.31 (6.58) ADHD (no reward) m = 27.72 (6.21) HC (reward) M = 25.13 (5.43) HC (no reward) m = 24.75 (3.63)	time reproduction task seven time intervals: 2 s, 6 s, 12 s, 24 s, 26 s, 48 s, 60 s time discrimination task discrimination between 1000ms and 1300ms intervals ANCOVA, sensitivity threshold, absolute discrepancy, accuracy coefficient	Behavioural data: Absolute discrepancy: ADHD < HC (*p* < 0.001) Effect of reward: n.s. Time discrimination: n.s. Effect of reward: n.s	η2 = 0.5
Nakashima et al. 2021 [79]	Time management, RCT	Examine the efficacy of a group cognitive training in time management in adult ADHD	N = 24 (adult ADHD, TAU, and intervention) ADHD Patients met DSM-IV Criteria	M = 39.11 (9.62) (whole sample)	Intervention: cognitive program, training of time management abilities (8 weeks) TAU: pharmacological and non-pharmacological treatment in an outpatient clinic Three follow-ups: 2, 4 and 8 months Outcome analyses: Sheehan disability scale, the CAARS and CGI-S Linear mixed effects models	Behavioural data: Intervention effect T1-T2 (gCBT) CAARS CBT > TAU (*p* < 0.001, CI 71.70–80.73) CGI (*p* < 0.001, CI 3.30–3.99) Sheehan (*p* < 0.001, CI 5.92–6.98) Intervention effect T1–T4 (gCBT) CAARS CBT>TAU (*p* < 0.001, CI 0.30–1.57) CGI (*p* < 0.001 CI 1.65–3.27) Sheehan (*p* < 0.001 CI 0.37–1.66)	d = 0.64 d = 2.75 d = 1.06 d = 0.95 d = 2.47 d = 1.02

Note ADHD: Attention deficit hyperactivity disorder; AE: Absolute error; RE: Relative error, s: second; PFC: prefrontal cortex, ACC: anterior cingulate cortex, DLPFC: dorsolateral prefrontal cortex, VLPFC: ventrolateral prefrontal cortex, DSM: Diagnostic and statistical manual of mental disorders; ICD-10: International classification of diseases, m: male, f: female, n.s.: non-significant, EEG: Electroencephalography, EF: executive functions, MEG: magnetoencephalography, SMA: supplementary motor area, ADS: Mean Absolute Discrepancy Score, ACS: Accuracy Coefficient Score, VBM: voxel-based morphometry, WM: working memory, STM: short-term memory.

## Data Availability

Not applicable.

## Supplementary Materials

The following supporting information can be downloaded at: https://www.mdpi.com/article/10.3390/ijerph20043098/s1, Scheme in text form.

## References

[1] Kessler R.C., Adler M.L.A., Barkley R., Biederman J., Conners C.K., Demler O., Faraone S.V., Greenhill L.L., Howes M.J., Secnik K. (2006). The Prevalence and Correlates of Adult ADHD in the United States: Results from the National Comorbidity Survey Replication. Am. J. Psychiatry.

[2] Schlack R., Hölling H., Kurth B.-M., Huss M. (2007). Die Prävalenz der Aufmerksamkeitsdefizit-/Hyperaktivitätsstörung (ADHS) bei Kindern und Jugendlichen in Deutschland. Erste Ergebnisse aus dem Kinder- und Jugendgesundheitssurvey (KiGGS). Bundesgesundheitsblatt Gesundh. Gesundh..

[3] Thapar A., Cooper M. (2016). Attention deficit hyperactivity disorder. Lancet.

[4] Bangma D.F., Koerts J., Fuermaier A.B.M., Mette C., Zimmermann M., Toussaint A.K., Tucha L., Tucha O. (2019). Financial decision-making in adults with ADHD. Neuropsychology.

[5] Bangma D.F., Tucha L., Fuermaier A.B.M., Tucha O., Koerts J. (2020). Financial decision-making in a community sample of adults with and without current symptoms of ADHD. PLoS ONE.

[6] Tucha O., Fuermaier A.B.M. (2021). Neuropsychological and real-life functioning of adults with ADHD. J. Neural Transm..

[7] Biederman J., Petty C.R., Clarke A., Lomedico A., Faraone S.V. (2011). Predictors of persistent ADHD: An 11-year follow-up study. J. Psychiatr. Res..

[8] Biederman J., Petty C.R., Evans M., Small J., Faraone S.V. (2010). How persistent is ADHD? A controlled 10-year follow-up study of boys with ADHD. Psychiatry Res..

[9] Faraone S.V., Banaschewski T., Coghill D., Zheng Y., Biederman J., Bellgrove M.A., Newcorn J.H., Gignac M., Al Saud N.M., Manor I. (2021). The World Federation of ADHD International Consensus Statement: 208 Evidence-based conclusions about the disorder. Neurosci. Biobehav. Rev..

[10] Fayyad J., Sampson N.A., Hwang I., Adamowski T., Aguilar-Gaxiola S., Al-Hamzawi A., Andrade L.H.S.G., Borges G., de Girolamo G., Florescu S. (2017). The descriptive epidemiology of DSM-IV Adult ADHD in the World Health Organization World Mental Health Surveys. ADHD Atten. Deficit Hyperact. Disord..

[11] Kooij J., Bijlenga D., Salerno L., Jaeschke R., Bitter I., Balázs J., Thome J., Dom G., Kasper S., Filipe C.N. (2018). Updated European Consensus Statement on diagnosis and treatment of adult ADHD. Eur. Psychiatry.

[12] Huss M., Hölling H., Kurth B.-M., Schlack R. (2008). How often are German children and adolescents diagnosed with ADHD? Prevalence based on the judgment of health care professionals: Results of the German health and examination survey (KiGGS). Eur. Child Adolesc. Psychiatry.

[13] Faraone S.V., Asherson P., Banaschewski T., Biederman J., Buitelaar J.K., Ramos-Quiroga J.A., Rohde L.A., Sonuga-Barke E.J.S., Tannock R., Franke B. (2015). Attention-deficit/hyperactivity disorder. Nat. Rev. Dis. Prim..

[14] Sharma A., Couture J. (2014). A Review of the Pathophysiology, Etiology, and Treatment of Attention-Deficit Hyperactivity Disorder (ADHD). Ann. Pharmacother..

[15] Das Banerjee T., Middleton F., Faraone S.V. (2007). Environmental risk factors for attention-deficit hyperactivity disorder. Acta Paediatr..

[16] Froehlich T.E., Anixt J.S., Loe I.M., Chirdkiatgumchai V., Kuan L., Gilman R.C. (2011). Update on Environmental Risk Factors for Attention-Deficit/Hyperactivity Disorder. Curr. Psychiatry Rep..

[17] Riglin L., Collishaw S., Thapar A.K., Dalsgaard S., Langley K., Smith G.D., Stergiakouli E., Maughan B., O’Donovan M.C., Thapar A. (2016). Association of Genetic Risk Variants With Attention-Deficit/Hyperactivity Disorder Trajectories in the General Population. JAMA Psychiatry.

[18] van Dyk L., Springer P., Kidd M., Steyn N., Solomons R., van Toorn R. (2014). Familial-Environmental Risk Factors in South African Children With Attention-Deficit Hyperactivity Disorder (ADHD): A Case-Control Study. J. Child Neurol..

[19] Palladino V.S., McNeill R., Reif A., Kittel-Schneider S. (2019). Genetic risk factors and gene–environment interactions in adult and childhood attention-deficit/hyperactivity disorder. Psychiatr. Genet..

[20] De Zeeuw P., Zwart F., Schrama R., Van Engeland H., Durston S. (2012). Prenatal exposure to cigarette smoke or alcohol and cerebellum volume in attention-deficit/hyperactivity disorder and typical development. Transl. Psychiatry.

[21] Barkley R.A. (2010). Differential Diagnosis of Adults with ADHD: The role of executive function and self-regulation. J. Clin. Psychiatry.

[22] Barkley R.A., Murphy K.R. (2010). Impairment in Occupational Functioning and Adult ADHD: The Predictive Utility of Executive Function (EF) Ratings Versus EF Tests. Arch. Clin. Neuropsychol..

[23] Cortese S., Coghill D. (2018). Twenty years of research on attention-deficit/hyperactivity disorder (ADHD): Looking back, looking forward. Évid. Based Ment. Health.

[24] Hoogman M., Muetzel R., Guimaraes J.P., Shumskaya E., Mennes M., Zwiers M.P., Jahanshad N., Sudre G., Wolfers T., Earl E.A. (2019). Brain Imaging of the Cortex in ADHD: A Coordinated Analysis of Large-Scale Clinical and Population-Based Samples. Am. J. Psychiatry.

[25] Mohamed S.M.H., Butzbach M., Fuermaier A.B.M., Weisbrod M., Aschenbrenner S., Tucha L., Tucha O. (2021). Basic and complex cognitive functions in Adult ADHD. PLoS ONE.

[26] Van Dessel J., Morsink S., Van der Oord S., Lemiere J., Moerkerke M., Grandelis M., Sonuga-Barke E., Danckaerts M. (2019). Waiting impulsivity: A distinctive feature of ADHD neuropsychology?. Child Neuropsychol..

[27] Gmehlin D., Fuermaier A.B.M., Walther S., Debelak R., Rentrop M., Westermann C., Sharma A., Tucha L., Koerts J., Tucha O. (2014). Intraindividual Variability in Inhibitory Function in Adults with ADHD—An Ex-Gaussian Approach. PLoS ONE.

[28] Ludyga S., Ishihara T. (2022). Brain structural changes and the development of interference control in children with ADHD: The predictive value of physical activity and body mass index. NeuroImage Clin..

[29] Mu S., Wu H., Zhang J., Chang C. (2022). Structural Brain Changes and Associated Symptoms of ADHD Subtypes in Children. Cereb. Cortex.

[30] Fuermaier A.B.M., Tucha L., Guo N., Mette C., Müller B.W., Scherbaum N., Tucha O. (2022). It Takes Time: Vigilance and Sustained Attention Assessment in Adults with ADHD. Int. J. Environ. Res. Public Health.

[31] Guo N., Fuermaier A.B.M., Koerts J., Tucha O., Scherbaum N., Müller B.W. (2022). Networks of Neuropsychological Functions in the Clinical Evaluation of Adult ADHD. Assessment.

[32] Mostert J.C., Hoogman M., Onnink A.M.H., van Rooij D., von Rhein D., van Hulzen K.J.E., Dammers J., Kan C.C., Buitelaar J.K., Norris D.G. (2018). Similar Subgroups Based on Cognitive Performance Parse Heterogeneity in Adults with ADHD and Healthy Controls. J. Atten. Disord..

[33] Fuermaier A.B.M., Tucha L., Koerts J., Aschenbrenner S., Kaunzinger I., Hauser J., Weisbrod M., Lange K.W., Tucha O. (2015). Cognitive impairment in adult ADHD—Perspective matters!. Neuropsychology.

[34] Barkley R.A. (1997). Behavioral inhibition, sustained attention, and executive functions: Constructing a unifying theory of ADHD. Psychol. Bull..

[35] Barkley R.A. (2013). Distinguishing Sluggish Cognitive Tempo From ADHD in Children and Adolescents: Executive Functioning, Impairment, and Comorbidity. J. Clin. Child Adolesc. Psychol..

[36] Brown T.E. (2008). ADD/ADHD and Impaired Executive Function in Clinical Practice. Curr. Psychiatry Rep..

[37] Sonuga-Barke E.J. (2003). The dual pathway model of AD/HD: An elaboration of neuro-developmental characteristics. Neurosci. Biobehav. Rev..

[38] Sonuga-Barke E.J. (2002). Psychological heterogeneity in AD/HD—A dual pathway model of behaviour and cognition. Behav. Brain Res..

[39] Sonuga-Barke E., Bitsakou P., Thompson M. (2010). Beyond the Dual Pathway Model: Evidence for the Dissociation of Timing, Inhibitory, and Delay-Related Impairments in Attention-Deficit/Hyperactivity Disorder. J. Am. Acad. Child Adolesc. Psychiatry.

[40] De Zeeuw P., Weusten J., Van Dijk S., Van Belle J., Durston S. (2012). Deficits in Cognitive Control, Timing and Reward Sensitivity Appear to be Dissociable in ADHD. PLoS ONE.

[41] Meck W.H. (2005). Neuropsychology of timing and time perception. Brain Cogn..

[42] Buhusi C.V., Meck W. (2005). What makes us tick? Functional and neural mechanisms of interval timing. Nat. Rev. Neurosci..

[43] Buhusi C.V., Meck W. (2009). Relativity Theory and Time Perception: Single or Multiple Clocks?. PLoS ONE.

[44] Meck W.H., Malapani C. (2004). Neuroimaging of interval timing. Cogn. Brain Res..

[45] Crystal J.D. (2001). Circadian time perception. J. Exp. Psychol. Anim. Behav. Process.

[46] Merchant H., de Lafuente V. (2014). Introduction to the Neurobiology of Interval Timing. Adv. Exp. Med. Biol..

[47] Meck W.H., Church R.M., Matell M. (2013). Hippocampus, time, and memory—A retrospective analysis. Behav. Neurosci..

[48] Meck W.H., Church R.M., Olton D.S. (2013). Hippocampus, time, and memory. Behav. Neurosci..

[49] Lalonde R., Hannequin D. (1999). The Neurobiological Basis of Time Estimation and Temporal Order. Rev. Neurosci..

[50] Lewis P.A., Miall R.C. (2003). Distinct systems for automatic and cognitively controlled time measurement: Evidence from neuroimaging. Curr. Opin. Neurobiol..

[51] Macar F., Lejeune H., Bonnet M., Ferrara A., Pouthas V., Vidal F., Maquet P. (2002). Activation of the supplementary motor area and of attentional networks during temporal processing. Exp. Brain Res..

[52] Buhusi C.V., Reyes M., Gathers C.-A., Oprisan S.A., Buhusi M. (2018). Inactivation of the Medial-Prefrontal Cortex Impairs Interval Timing Precision, but Not Timing Accuracy or Scalar Timing in a Peak-Interval Procedure in Rats. Front. Integr. Neurosci..

[53] Kim J., Ghim J.-W., Lee J.H., Jung M.W., Branch S.Y., Goertz R.B., Sharpe A.L., Pierce J., Roy S., Ko D. (2013). Neural Correlates of Interval Timing in Rodent Prefrontal Cortex. J. Neurosci..

[54] Valera E.M., Faraone S.V., Biederman J., Poldrack R.A., Seidman L.J. (2005). Functional neuroanatomy of working memory in adults with attention-deficit/hyperactivity disorder. Biol. Psychiatry.

[55] Breska A., Ivry R.B. (2016). Taxonomies of timing: Where does the cerebellum fit in?. Curr. Opin. Behav. Sci..

[56] Petter E.A., Lusk N.A., Hesslow G., Meck W.H. (2016). Interactive roles of the cerebellum and striatum in sub-second and supra-second timing: Support for an initiation, continuation, adjustment, and termination (ICAT) model of temporal processing. Neurosci. Biobehav. Rev..

[57] Noreika V., Falter C.M., Rubia K. (2013). Timing deficits in attention-deficit/hyperactivity disorder (ADHD): Evidence from neurocognitive and neuroimaging studies. Neuropsychologia.

[58] Coghill D., Toplak M., Rhodes S., Adamo N., Banaschewski T., Zuddas D.C.A. (2018). Cognitive functioning in ADHD: Inhibition, memory, temporal discounting, decision-making, timing and reaction time variability. Oxford Textbook of Attention Deficit Hyperactivity Disorder.

[59] Zakay D. (2015). The temporal-relevance temporal-uncertainty model of prospective duration judgment. Conscious. Cogn..

[60] Claessens B.J., van Eerde W., Rutte C.G., Roe R.A. (2007). A review of the time management literature. Pers. Rev..

[61] Nejati V., Yazdani S. (2020). Time perception in children with attention deficit–hyperactivity disorder (ADHD): Does task matter? A meta-analysis study. Child Neuropsychol..

[62] Zheng Q., Wang X., Chiu K.Y., Shum K.K.-M. (2022). Time Perception Deficits in Children and Adolescents with ADHD: A Meta-analysis. J. Atten. Disord..

[63] Barkley R.A., Fischer M. (2019). Time Reproduction Deficits at Young Adult Follow-Up in Childhood ADHD: The Role of Persistence of Disorder and Executive Functioning. Dev. Neuropsychol..

[64] Valko L., Schneider G., Doehnert M., Müller U., Brandeis D., Steinhausen H.-C., Drechsler R. (2010). Time processing in children and adults with ADHD. J. Neural Transm..

[65] Smith A., Taylor E., Rogers J.W., Newman S., Rubia K. (2002). Evidence for a pure time perception deficit in children with ADHD. J. Child Psychol. Psychiatry.

[66] Yang B., Chan R.C., Zou X., Jing J., Mai J., Li J. (2007). Time perception deficit in children with ADHD. Brain Res..

[67] Barkley R.A., Murphy K.R., Bush T. (2001). Time perception and reproduction in young adults with attention deficit hyperactivity disorder. Neuropsychology.

[68] Toplak M., Rucklidge J., Hetherington R., John S., Tannock R. (2003). Time perception deficits in attention-deficit/ hyperactivity disorder and comorbid reading difficulties in child and adolescent samples. J. Child Psychol. Psychiatry.

[69] Hart H., Radua J., Mataix-Cols D., Rubia K. (2012). Meta-analysis of fMRI studies of timing in attention-deficit hyperactivity disorder (ADHD). Neurosci. Biobehav. Rev..

[70] Cohen J. (1988). Statistical Power Analysis for the Behavioral Sciences.

[71] Pretus C., Picado M., Ramos-Quiroga A., Carmona S., Richarte V., Fauquet J., Vilarroya O. (2020). Presence of Distractor Improves Time Estimation Performance in an Adult ADHD Sample. J. Atten. Disord..

[72] Fontes R.M., Marinho V., Carvalho V., Rocha K., Magalhães F., Moura I., Ribeiro P., Velasques B., Cagy M., Gupta D.S. (2020). Time estimation exposure modifies cognitive aspects and cortical activity of attention deficit hyperactivity disorder adults. Int. J. Neurosci..

[73] Mostert J.C., Onnink A.M.H., Klein M., Dammers J., Harneit A., Schulten T., van Hulzen K.J., Kan C.C., Slaats-Willemse D., Buitelaar J.K. (2015). Cognitive heterogeneity in adult attention deficit/hyperactivity disorder: A systematic analysis of neuropsychological measurements. Eur. Neuropsychopharmacol..

[74] Wilson T.W., Heinrichs-Graham E., White M.L., Knott N.L., Wetzel M.W. (2013). Estimating the passage of minutes: Deviant oscillatory frontal activity in medicated and unmedicated ADHD. Neuropsychology.

[75] Dölek G.T., Ozel-Kizil E.T., Bastug G., Baran Z., Colak B. (2021). Impaired auditory and visual time reproduction in adult patients with attention deficit-hyperactivity disorder. J. Clin. Exp. Neuropsychol..

[76] Pironti V.A., Lai M.-C., Morein-Zamir S., Müller U., Bullmore E.T., Sahakian B.J. (2016). Temporal reproduction and its neuroanatomical correlates in adults with attention deficit hyperactivity disorder and their unaffected first-degree relatives. Psychol. Med..

[77] Mette C., Grabemann M., Zimmermann M., Strunz L., Scherbaum N., Wiltfang J., Kis B. (2015). No Clear Association between Impaired Short-Term or Working Memory Storage and Time Reproduction Capacity in Adult ADHD Patients. PLoS ONE.

[78] Marx I., Höpcke C., Berger C., Wandschneider R., Herpertz S.C. (2013). The Impact of Financial Reward Contingencies on Cognitive Function Profiles in Adult ADHD. PLoS ONE.

[79] Nakashima M., Inada N., Tanigawa Y., Yamashita M., Maeda E., Kouguchi M., Sarad Y., Yano H., Ikari K., Kuga H. (2022). Efficacy of Group Cognitive Behavior Therapy Targeting Time Management for Adults with Attention Deficit/Hyperactivity Disorder in Japan: A Randomized Control Pilot Trial. J. Atten. Disord..

[80] Zangbar H.S., Ghadiri T., Vafaee M.S., Kalan A.E., Fallahi S., Ghorbani M., Shahabi P. (2020). Theta Oscillations through Hippocampal/Prefrontal Pathway: Importance in Cognitive Performances. Brain Connect..

[81] Love T.M. (2014). Oxytocin, motivation and the role of dopamine. Pharmacol. Biochem. Behav..

[82] De Leonibus E., Verheij M.M.M., Mele A., Cools A. (2006). Distinct kinds of novelty processing differentially increase extracellular dopamine in different brain regions. Eur. J. Neurosci..

[83] Stern A., Malik E., Pollak Y., Bonne O., Maeir A. (2016). The Efficacy of Computerized Cognitive Training in Adults with ADHD: A Randomized Controlled Trial. J. Atten. Disord..

[84] Hass J., Durstewitz D. (2014). Neurocomputational Models of Time Perception. Adv. Exp. Med. Biol..

[85] Clément A., Droit-Volet S. (2006). Counting in a time discrimination task in children and adults. Behav. Process..

[86] Mowlem F.D., Rosenqvist M.A., Martin J., Lichtenstein P., Asherson P., Larsson H. (2019). Sex differences in predicting ADHD clinical diagnosis and pharmacological treatment. Eur. Child Adolesc. Psychiatry.

[87] Kallweit C., Paucke M., Strauß M., Exner C. (2020). Cognitive deficits and psychosocial functioning in adult ADHD: Bridging the gap between objective test measures and subjective reports. J. Clin. Exp. Neuropsychol..

[88] Mohammadi M.-R., Zarafshan H., Khaleghi A., Ahmadi N., Hooshyari Z., Mostafavi S.-A., Ahmadi A., Alavi S.-S., Shakiba A., Salmanian M. (2021). Prevalence of ADHD and Its Comorbidities in a Population-Based Sample. J. Atten. Disord..

[89] Droit-Volet S. (2013). Time perception, emotions and mood disorders. J. Physiol..

