# Supplementary material for: Time Perception in Adult ADHD: Findings from a Decade—A Review
Source: Int J Environ Res Public Health. 2023 Feb 10;20(4):3098. doi: 10.3390/ijerph20043098 (PMC9962130; doi:10.3390/ijerph20043098)
Supplement: Supplementary file 1 [file ijerph-20-03098-s001.zip › ijerph-2124918-supplementary.pdf]

Supplementary online material

In addition, according to the literature the following variables were derived and defined as inclusion criteria for the present narrative review: firstly, papers had to be written in English or German and had to be listed in the databases „PubMed“, „Medline“ and „PSYNDEX“. Secondly, original research articles, clinical trials, meta-analyses were included in the present review. Thirdly, a period from 2012-2022 was defined as the range for the literature search. Further, adults with ADHD (>18 years) with a primary diagnosis of ADHD according to DSM-IV, DSM-5 or ICD-10 were included. Neither ADHD subtype presentation, gender/sex, comorbidities nor IQ restricted the search strategy. The following exclusion criteria were derived from the literature and defined for the present review: studies not including ADHD and time perception as described under inclusion criteria, studies including *only* children and adolescents, publications not subject to peer review, non-English or German papers, case studies, reviews, studies describing only psychiatric comorbidities and pharmacological trials and studies older than 10 years.

The following databases were searched using the search strategy copied below

Pubmed

“adult ADHD” and “time deficit”; “adult ADHD” and “timing deficit”; “adult ADHD” and “time perception”; “adult ADHD” and “time estimation”; “adult ADHD” and “time reproduction”; “adult ADHD” and “time production”; “adult ADHD” and “duration discrimination”; “adult ADHD” and “time management”; “adult ADHD” and “time processing”; “adult ADHD” and “time”; “adults” and “attention deficit hyperactivity

disorder” and “time deficit”; “adults” and “attention deficit hyperactivity disorder” and “timing deficit”; “adults” and “attention deficit hyperactivity disorder” and “time perception”; “adults” and “attention deficit hyperactivity disorder” and “time estimation”; “adults” and “attention deficit hyperactivity disorder” and “time reproduction”; “adults” and “attention deficit hyperactivity disorder” and “time production”; “adults” and “attention deficit hyperactivity disorder” and “duration discrimination”; “adults” and “attention deficit hyperactivity disorder” and “time management”; “adults” and “attention deficit hyperactivity disorder” and “time processing”; “adults” and “attention deficit hyperactivity disorder” and “time”

“adult ADHD” and “time deficit” n=1; “adult ADHD” and “timing deficit” n=1; “adult ADHD” and “time perception” n=9; “adult ADHD” and “time estimation” n=3; “adult ADHD” and “time reproduction” n=5; “adult ADHD” and “time production” n=0; “adult ADHD” and “duration discrimination” n=0; “adult ADHD” and “time management” n=4; “adult ADHD” and “time processing” n=2; “adult ADHD” and “time” n=175; Total n=200

“adults” and “attention deficit hyperactivity disorder” and “time deficit” n=0; “adults” and “attention deficit hyperactivity disorder” and “timing deficit” n=0; “adults” and “attention deficit hyperactivity disorder” and “time perception” n=20; “adults” and “attention deficit hyperactivity disorder” and “time estimation” n=8; “adults” and “attention deficit hyperactivity disorder” and “time reproduction” n=6; “adults” and “attention deficit hyperactivity disorder” and “time production” n=0; “adults” and “attention deficit hyperactivity disorder” and “duration discrimination” n=2; “adults” and “attention deficit hyperactivity disorder” and “time management” n=16; “adults” and “attention deficit hyperactivity disorder” and “time processing” n=4; “adults” and “attention deficit hyperactivity disorder” and “time” n= 302; Total n=358

Medline

“adult ADHD” and “time deficit”; “adult ADHD” and “timing deficit”; “adult ADHD” and “time perception”; “adult ADHD” and “time estimation”; “adult ADHD” and “time reproduction”; “adult ADHD” and “time production”; “adult ADHD” and “duration discrimination”; “adult ADHD” and “time management”; “adult ADHD” and “time processing”; “adult ADHD” and “time”; “adults” and “attention deficit hyperactivity disorder” and “time deficit”; “adults” and “attention deficit hyperactivity disorder” and “timing deficit”; “adults” and “attention deficit hyperactivity disorder” and “time perception”; “adults” and “attention deficit hyperactivity disorder” and “time estimation”; “adults” and “attention deficit hyperactivity disorder” and “time reproduction”; “adults” and “attention deficit hyperactivity disorder” and “time production”; “adults” and “attention deficit hyperactivity disorder” and “duration discrimination”; “adults” and “attention deficit hyperactivity disorder” and “time management”; “adults” and “attention deficit hyperactivity disorder” and “time processing”; “adults” and “attention deficit hyperactivity disorder” and “time”

“adult ADHD” and “time deficit”; n=1; “adult ADHD” and “timing deficit” n=0; “adult ADHD” and “time perception” n=8; “adult ADHD” and “time estimation” n=1; “adult ADHD” and “time reproduction” n=5; “adult ADHD” and “time production” n=0; “adult ADHD” and “duration discrimination” n=0; “adult ADHD” and “time management” n=3; “adult ADHD” and “time processing” n=2; “adult ADHD” and “time” n=175; Total n=195

“adults” and “attention deficit hyperactivity disorder” and “time deficit”; n=0; “adults” and “attention deficit hyperactivity disorder” and “timing deficit” n=1; “adults” and “attention deficit hyperactivity disorder” and “time perception” n=13; “adults” and “attention deficit hyperactivity disorder” and “time estimation” n=4; “adults” and “attention deficit hyperactivity disorder” and “time reproduction” n=3; “adults” and

“attention deficit hyperactivity disorder” and “time production” n=0; “adults” and “attention deficit hyperactivity disorder” and “duration discrimination” n=1; “adults” and “attention deficit hyperactivity disorder” and “time management” n=1; “adults” and “attention deficit hyperactivity disorder” and “time processing” n=3; “adults” and “attention deficit hyperactivity disorder” and “time” n=302; Total n=328

PSYNDEX

“adult ADHD” and “time deficit”; “adult ADHD” and “timing deficit”; “adult ADHD” and “time perception”; “adult ADHD” and “time estimation”; “adult ADHD” and “time reproduction”; “adult ADHD” and “time production”; “adult ADHD” and “duration discrimination”; “adult ADHD” and “time management”; “adult ADHD” and “time processing”; “adult ADHD” and “time”; “adults” and “attention deficit hyperactivity disorder” and “time deficit”; “adults” and “attention deficit hyperactivity disorder” and “timing deficit”; “adults” and “attention deficit hyperactivity disorder” and “time perception”; “adults” and “attention deficit hyperactivity disorder” and “time estimation”; “adults” and “attention deficit hyperactivity disorder” and “time reproduction”; “adults” and “attention deficit hyperactivity disorder” and “time production”; “adults” and “attention deficit hyperactivity disorder” and “duration discrimination”; “adults” and “attention deficit hyperactivity disorder” and “time management”; “adults” and “attention deficit hyperactivity disorder” and “time processing”; “adults” and “attention deficit hyperactivity disorder” and “time”

“adult ADHD” and “time deficit” n=0; “adult ADHD” and “timing deficit” n=0; “adult ADHD” and “time perception” n=0; “adult ADHD” and “time estimation” n=1; “adult ADHD” and “time reproduction” n=1; “adult ADHD” and “time production” n=0; “adult

ADHD" and "duration discrimination" n=0; "adult ADHD" and "time management" n=0; "adult ADHD" and "time processing" n=0; "adult ADHD" and "time" n=30;Total n=41

"adults" and "attention deficit hyperactivity disorder" and "time deficit"; n=0; "adults" and "attention deficit hyperactivity disorder" and "timing deficit" n=0; "adults" and "attention deficit hyperactivity disorder" and "time perception" n=1; "adults" and "attention deficit hyperactivity disorder" and "time estimation" n=1; "adults" and "attention deficit hyperactivity disorder" and "time reproduction" n=1; "adults" and "attention deficit hyperactivity disorder" and "time production" n=0; "adults" and "attention deficit hyperactivity disorder" and "duration discrimination" n=0; "adults" and "attention deficit hyperactivity disorder" and "time management" n=1; "adults" and "attention deficit hyperactivity disorder" and "time processing" n=0;"adults" and "attention deficit hyperactivity disorder" and "time" n=60; Total n=64
